# Supplementary material for: Thyroid Hormone Receptor β Knockdown Reduces Suppression of Progestins by Activating the mTOR Pathway in Endometrial Cancer Cells
Source: Int J Mol Sci. 2022 Oct 19;23(20):12517. doi: 10.3390/ijms232012517 (PMC9604373; doi:10.3390/ijms232012517)
Supplement: Supplementary file 1 [file ijms-23-12517-s001.zip › ijms-1889932 Supplementary.pdf]

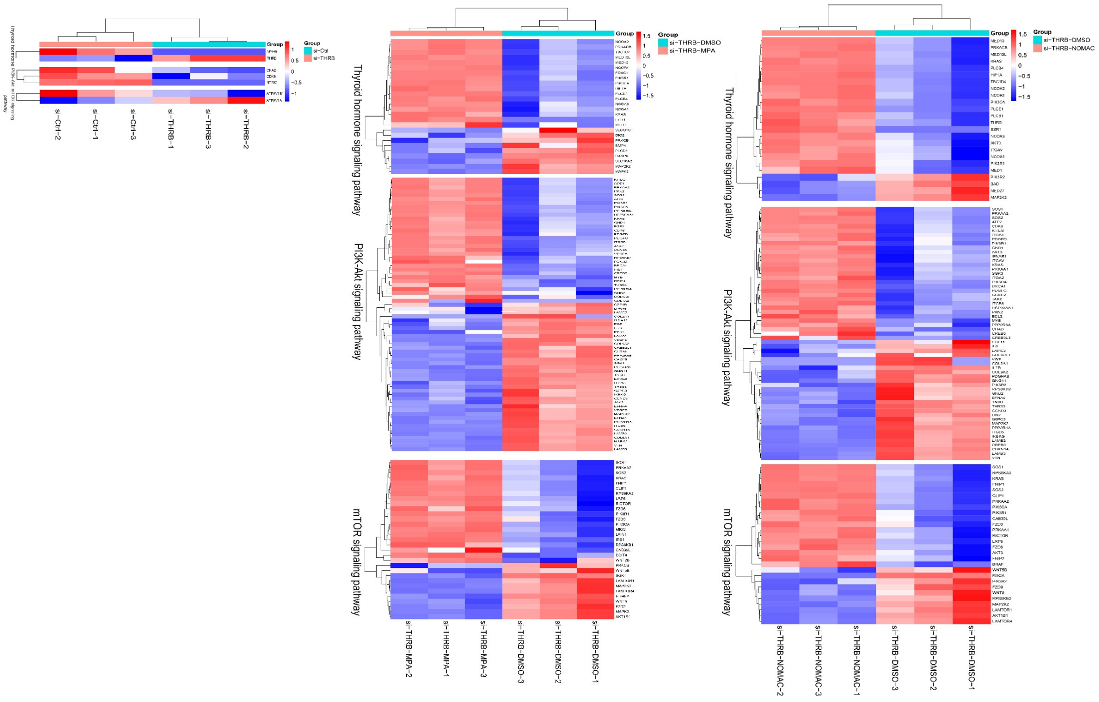

(**A**) (B) (**C**)

Figure S1. Heatmaps of DEGs associated with thyroid hormone, PI3K-Akt, and mTOR signaling pathways. (**A**) si-Ctrl-RL95-2 and si-THRB-RL95-2 cells. (**B**) si-THRB-DMSO-RL95-2 and si-THRB-MPA-RL95-2 cells. (**C**) si-THRB-DMSO-RL95-2 and si-THRB-NOMAC-RL95-2 cells. THRB, thyroid hormone receptor beta; si-Ctrl, negative control treated with siRNA solvent; si-THRB, silenced THRB; MPA, medroxyprogesterone acetate; NOMAc, nomegestrol acetate; DEGs, differentially expressed genes.

Table S1. List of differentially expressed genes correlated with thyroid hormone, PI3K-Akt and mTOR signaling pathway between si-Ctrl *vs* si-THRB treatment RL95-2 cells.

**Pathway name**	**Gene**	**log2FoldChange**	***P* value**	**FDR**
Thyroid hormone signaling pathway	THRB	1.180189231	6.81E-09	8.62E-06
Thyroid hormone signaling pathway	MYH6	-2.111504492	0.046155276	0.444464178
PI3K-Akt signaling pathway	CHAD	1.075691143	0.011700901	0.247669069
PI3K-Akt signaling pathway	CDK6	1.116737417	5.47E-06	0.002304243
PI3K-Akt signaling pathway	NTRK1	2.582805324	0.010882694	0.23953765
mTOR signaling pathway	ATP6V1A	1.155432405	9.68E-07	0.000524658
mTOR signaling pathway	ATP6V1B1	-1.213437595	0.001220544	0.08388027

FDR represented for false discovery rate. Log2FoldChange represented for the fold changes of the log2 ratio of gene expression in si-Ctrl to that in si-THRB treatment RL95-2 cells. *P* value was used to indicate the significance level of the differential gene between si-Ctrl *vs* si-THRB treatment RL95-2 cells.

Table S2. List of differentially expressed genes correlated to thyroid hormone, PI3K-Akt and mTOR signaling pathway between si-THRB-DMSO *vs* si-THRB-MPA treatment RL95-2 cells.

Pathway name	Gene	log2FoldChange	*P* value	FDR
Thyroid hormone signaling pathway	MED13L	-1.309592398	1.50E-11	2.68E-10
Thyroid hormone signaling pathway	PRKCB	2.155251754	2.22E-06	1.56E-05
Thyroid hormone signaling pathway	SLC16A2	1.353875019	9.91E-08	8.83E-07
Thyroid hormone signaling pathway	FOXO1	-1.270636644	6.04E-13	1.38E-11
Thyroid hormone signaling pathway	SLCO1C1	1.159994925	0.008493799	0.029677187
Thyroid hormone signaling pathway	NCOR1	-1.395921153	1.66E-14	4.92E-13
Thyroid hormone signaling pathway	DIO2	1.362939273	4.73E-10	6.46E-09
Thyroid hormone signaling pathway	NCOA1	-1.216976853	2.76E-07	2.28E-06
Thyroid hormone signaling pathway	PIK3R1	-1.62986712	1.49E-12	3.17E-11
Thyroid hormone signaling pathway	MAP2K2	1.02856524	1.86E-13	4.56E-12
Thyroid hormone signaling pathway	PIK3CA	-1.317099694	2.84E-17	1.34E-15
Thyroid hormone signaling pathway	MED1	-1.221463004	0.005697726	0.020754592
Thyroid hormone signaling pathway	HIF1A	-1.632798172	1.24E-24	1.76E-22
Thyroid hormone signaling pathway	PLCE1	-2.227678153	2.92E-50	4.04E-47
Thyroid hormone signaling pathway	NCOA3	-1.08104763	1.71E-07	1.46E-06
Thyroid hormone signaling pathway	ESR1	-2.440679717	0.021772669	0.068138776
Thyroid hormone signaling pathway	PRKACB	-1.26926402	3.25E-12	6.53E-11
Thyroid hormone signaling pathway	PLCB4	-1.443576357	6.41E-12	1.21E-10
Thyroid hormone signaling pathway	PLCD1	1.204670177	1.52E-05	9.24E-05
Thyroid hormone signaling pathway	MED13	-1.969537701	5.68E-16	2.19E-14
Thyroid hormone signaling pathway	CASP9	1.092076858	3.65E-24	4.76E-22
Thyroid hormone signaling pathway	NCOA2	-1.433331916	1.79E-14	5.28E-13
Thyroid hormone signaling pathway	KRAS	-1.141224314	1.51E-09	1.88E-08
Thyroid hormone signaling pathway	BMP4	1.134450067	0.000226268	0.001108432
Thyroid hormone signaling pathway	TBC1D4	-1.314311399	2.09E-14	6.06E-13
Thyroid hormone signaling pathway	MAPK3	1.286466993	9.93E-19	5.96E-17
PI3K-Akt signaling pathway	CREB3L1	1.828536898	7.52E-14	1.98E-12
PI3K-Akt signaling pathway	ITGA3	1.031615954	3.26E-13	7.71E-12
PI3K-Akt signaling pathway	BRCA1	-1.924630902	1.03E-40	7.86E-38
PI3K-Akt signaling pathway	TNXB	1.571667842	7.48E-12	1.40E-10
PI3K-Akt signaling pathway	IRS1	-1.26680742	3.45E-20	2.55E-18
PI3K-Akt signaling pathway	CHRM1	3.113400147	0.000727219	0.003230258
PI3K-Akt signaling pathway	GNB4	-1.379084001	2.89E-09	3.41E-08
PI3K-Akt signaling pathway	EIF4E2	1.042819835	6.38E-17	2.85E-15
PI3K-Akt signaling pathway	PDGFRB	2.086674519	1.14E-20	9.05E-19
PI3K-Akt signaling pathway	JAK2	-1.676770164	1.54E-15	5.54E-14
PI3K-Akt signaling pathway	VEGFA	-1.182279573	7.61E-09	8.37E-08
PI3K-Akt signaling pathway	LAMA1	1.315843008	1.02E-10	1.58E-09
PI3K-Akt signaling pathway	CCNE2	-1.14968059	8.39E-11	1.32E-09
PI3K-Akt signaling pathway	GNG11	1.224778465	1.03E-20	8.25E-19
PI3K-Akt signaling pathway	CDKN1A	1.895977072	3.11E-42	2.49E-39
PI3K-Akt signaling pathway	PIK3R1	-1.62986712	1.49E-12	3.17E-11
PI3K-Akt signaling pathway	COL1A2	-2.125206073	0.018387678	0.05871461
PI3K-Akt signaling pathway	ITGB5	1.132038919	4.59E-19	2.89E-17
PI3K-Akt signaling pathway	VEGFB	1.240037572	1.84E-13	4.52E-12
PI3K-Akt signaling pathway	MYB	-1.613919212	4.99E-10	6.78E-09
PI3K-Akt signaling pathway	SOS2	-1.349515306	1.19E-11	2.17E-10
PI3K-Akt signaling pathway	EFNA1	1.293203532	8.76E-14	2.28E-12

PI3K-Akt signaling pathway	VEGFC	1.000740629	9.30E-08	8.33E-07
PI3K-Akt signaling pathway	MAP2K2	1.02856524	1.86E-13	4.56E-12
PI3K-Akt signaling pathway	PKN2	-1.514800802	8.72E-14	2.27E-12
PI3K-Akt signaling pathway	PRKAA2	-1.328735508	1.74E-11	3.06E-10
PI3K-Akt signaling pathway	DDIT4	-1.18020239	1.08E-21	9.91E-20
PI3K-Akt signaling pathway	PIK3CA	-1.317099694	2.84E-17	1.34E-15
PI3K-Akt signaling pathway	FGF2	-1.208105893	1.25E-08	1.33E-07
PI3K-Akt signaling pathway	IL7R	1.554608777	7.18E-08	6.58E-07
PI3K-Akt signaling pathway	ITGA11	1.938016937	1.90E-05	0.000113823
PI3K-Akt signaling pathway	COL6A3	-2.280877059	0.041057591	0.117768221
PI3K-Akt signaling pathway	COL6A1	1.13438026	8.20E-17	3.61E-15
PI3K-Akt signaling pathway	KITLG	-1.304076853	8.04E-11	1.27E-09
PI3K-Akt signaling pathway	PPP2R5E	-1.107066057	3.04E-13	7.22E-12
PI3K-Akt signaling pathway	FOXO3	-1.064627785	7.30E-07	5.61E-06
PI3K-Akt signaling pathway	CSF1R	2.207740767	0.046877485	0.131941662
PI3K-Akt signaling pathway	COL9A2	1.951651778	3.03E-10	4.29E-09
PI3K-Akt signaling pathway	LAMB3	1.638964099	1.78E-33	7.12E-31
PI3K-Akt signaling pathway	IKBKG	1.015355581	1.19E-09	1.51E-08
PI3K-Akt signaling pathway	EGF	1.170212614	8.45E-06	5.38E-05
PI3K-Akt signaling pathway	PDGFD	-1.006908303	0.000210839	0.00104071
PI3K-Akt signaling pathway	SGK1	1.070012249	9.30E-23	9.98E-21
PI3K-Akt signaling pathway	ITGB8	-1.250297382	7.19E-10	9.49E-09
PI3K-Akt signaling pathway	GNG2	-2.266355071	0.009860764	0.03391689
PI3K-Akt signaling pathway	PPP2R5B	1.203313675	2.23E-12	4.61E-11
PI3K-Akt signaling pathway	THBS4	-1.559539858	0.005928208	0.021506774
PI3K-Akt signaling pathway	ATF2	-1.559525965	4.82E-11	7.86E-10
PI3K-Akt signaling pathway	LAMB2	1.243310759	2.01E-22	2.08E-20
PI3K-Akt signaling pathway	CASP9	1.092076858	3.65E-24	4.76E-22
PI3K-Akt signaling pathway	LAMC2	1.103332763	0.0386654	0.111928091
PI3K-Akt signaling pathway	PPP2R3A	-1.079486886	7.74E-05	0.000415116
PI3K-Akt signaling pathway	SOS1	-1.560416883	5.44E-12	1.04E-10
PI3K-Akt signaling pathway	THBS3	1.179202072	1.73E-15	6.16E-14
PI3K-Akt signaling pathway	EFNA4	1.35076033	5.41E-09	6.07E-08
PI3K-Akt signaling pathway	KRAS	-1.141224314	1.51E-09	1.88E-08
PI3K-Akt signaling pathway	CDK6	-1.326507148	4.81E-09	5.44E-08
PI3K-Akt signaling pathway	PCK1	3.482558521	0.000190554	0.00094919
PI3K-Akt signaling pathway	VTN	2.52969836	2.62E-53	5.33E-50
PI3K-Akt signaling pathway	CCND3	1.093360821	2.59E-11	4.44E-10
PI3K-Akt signaling pathway	CREB5	-1.430621924	2.62E-08	2.63E-07
PI3K-Akt signaling pathway	HSP90AA1	-1.143361087	4.68E-16	1.83E-14
PI3K-Akt signaling pathway	PPP2R1A	1.132525859	1.80E-17	8.93E-16
PI3K-Akt signaling pathway	LPAR5	1.172608387	0.041261499	0.118264084
PI3K-Akt signaling pathway	COL2A1	1.981298349	0.038427644	0.111334987
PI3K-Akt signaling pathway	MAPK3	1.286466993	9.93E-19	5.96E-17
PI3K-Akt signaling pathway	JAK3	1.013229046	1.26E-08	1.34E-07
PI3K-Akt signaling pathway	G6PC3	1.083080303	1.11E-08	1.19E-07
PI3K-Akt signaling pathway	PDGFC	-1.310630079	8.68E-16	3.28E-14
PI3K-Akt signaling pathway	RPS6KB1	-1.17503114	3.31E-10	4.64E-09
mTOR signaling pathway	PRKCB	2.155251754	2.22E-06	1.56E-05
mTOR signaling pathway	RICTOR	-1.447240613	0.00156812	0.006511051
mTOR signaling pathway	IRS1	-1.26680742	3.45E-20	2.55E-18
mTOR signaling pathway	EIF4E2	1.042819835	6.38E-17	2.85E-15

mTOR signaling pathway	WNT6	2.355149174	1.00E-19	6.92E-18
mTOR signaling pathway	LAMTOR4	1.239779874	2.41E-13	5.83E-12
mTOR signaling pathway	PIK3R1	-1.62986712	1.49E-12	3.17E-11
mTOR signaling pathway	FZD3	-1.068631313	7.04E-05	0.000381199
mTOR signaling pathway	FZD2	1.037312829	2.80E-10	4.00E-09
mTOR signaling pathway	SOS2	-1.349515306	1.19E-11	2.17E-10
mTOR signaling pathway	MAP2K2	1.02856524	1.86E-13	4.56E-12
mTOR signaling pathway	PRKAA2	-1.328735508	1.74E-11	3.06E-10
mTOR signaling pathway	DDIT4	-1.18020239	1.08E-21	9.91E-20
mTOR signaling pathway	PIK3CA	-1.317099694	2.84E-17	1.34E-15
mTOR signaling pathway	WNT2B	-1.886940071	0.001448234	0.006056993
mTOR signaling pathway	AKT1S1	1.300678809	1.91E-18	1.10E-16
mTOR signaling pathway	FZD6	-1.312262144	1.97E-08	2.02E-07
mTOR signaling pathway	MIOS	-1.204364032	1.21E-11	2.20E-10
mTOR signaling pathway	WNT5B	1.868709439	0.001515721	0.006314122
mTOR signaling pathway	SGK1	1.070012249	9.30E-23	9.98E-21
mTOR signaling pathway	LPIN1	-2.172586698	3.56E-23	4.10E-21
mTOR signaling pathway	SOS1	-1.560416883	5.44E-12	1.04E-10
mTOR signaling pathway	FNIP1	-1.544751014	6.05E-12	1.15E-10
mTOR signaling pathway	KRAS	-1.141224314	1.51E-09	1.88E-08
mTOR signaling pathway	LRP6	-1.267335266	3.22E-10	4.53E-09
mTOR signaling pathway	LAMTOR1	1.087518013	3.69E-12	7.32E-11
mTOR signaling pathway	MAPK3	1.286466993	9.93E-19	5.96E-17
mTOR signaling pathway	CAB39L	-1.101296683	0.00197946	0.00802018
mTOR signaling pathway	CLIP1	-1.001327712	1.97E-09	2.41E-08
mTOR signaling pathway	RPS6KA3	-1.202947839	3.79E-09	4.37E-08
mTOR signaling pathway	RPS6KB1	-1.17503114	3.31E-10	4.64E-09

FDR represented for false discovery rate. Log2FoldChange represented for the fold changes of the log2 ratio of gene expression in si-THRB-DMSO to that in si-THRB-MPA treatment RL95-2 cells. *P* value was used to indicate the significance level of the differential gene between si-THRB-DMSO *vs* si-THRB-MPA treatment RL95-2 cells.

Table S3. List of differentially expressed genes associated with thyroid hormone, PI3K-Akt and mTOR signaling pathway between si-THRB-DMSO *vs* si-THRB-NOMAC treatment RL95-2 cells.

Pathway name	Gene	log2FoldChange	*P* value	FDR
Thyroid hormone signaling pathway	MED13L	-1.355148267	4.41E-12	1.16E-10
Thyroid hormone signaling pathway	THRB	-1.168124176	2.32E-09	3.30E-08
Thyroid hormone signaling pathway	MED27	1.052923174	2.01E-09	2.91E-08
Thyroid hormone signaling pathway	NCOR1	-1.341813614	9.87E-13	2.98E-11
Thyroid hormone signaling pathway	NCOA1	-1.045286951	1.58E-05	0.000102851
Thyroid hormone signaling pathway	PIK3R1	-1.238014643	2.21E-07	2.07E-06
Thyroid hormone signaling pathway	AKT3	-1.108506573	2.78E-06	2.10E-05
Thyroid hormone signaling pathway	MAP2K2	1.203248028	5.26E-16	3.50E-14
Thyroid hormone signaling pathway	PIK3CA	-1.411174231	4.71E-19	6.39E-17
Thyroid hormone signaling pathway	MED1	-1.383287986	1.59E-08	1.90E-07
Thyroid hormone signaling pathway	HIF1A	-1.455714036	2.78E-20	4.72E-18
Thyroid hormone signaling pathway	PLCE1	-1.939040533	2.46E-37	5.91E-34
Thyroid hormone signaling pathway	NCOA3	-1.208311254	6.05E-09	7.92E-08
Thyroid hormone signaling pathway	ESR1	-3.574433593	8.35E-05	0.000473964
Thyroid hormone signaling pathway	PRKACB	-1.251479377	6.11E-12	1.57E-10
Thyroid hormone signaling pathway	PLCB4	-1.377225186	4.00E-11	8.47E-10

Thyroid hormone signaling pathway	MED13	-1.998823522	4.89E-16	3.29E-14
Thyroid hormone signaling pathway	PIK3R2	1.154260485	8.09E-08	8.35E-07
Thyroid hormone signaling pathway	NCOA2	-1.469384803	8.22E-15	3.99E-13
Thyroid hormone signaling pathway	KRAS	-1.217668059	3.46E-11	7.44E-10
Thyroid hormone signaling pathway	ITGAV	-1.029953061	2.90E-06	2.18E-05
Thyroid hormone signaling pathway	BAD	1.195966943	1.29E-13	4.76E-12
Thyroid hormone signaling pathway	TBC1D4	-1.223938012	8.27E-13	2.54E-11
Thyroid hormone signaling pathway	PLCB1	-1.239086562	2.42E-11	5.41E-10
PI3K-Akt signaling pathway	CREB3L1	1.043242383	0.000147404	0.000791499
PI3K-Akt signaling pathway	BRCA1	-1.848148195	1.23E-38	3.85E-35
PI3K-Akt signaling pathway	BCL2	-1.027908772	1.37E-10	2.57E-09
PI3K-Akt signaling pathway	TNXB	1.655966684	4.11E-11	8.67E-10
PI3K-Akt signaling pathway	GNB4	-1.282905358	4.92E-08	5.30E-07
PI3K-Akt signaling pathway	PDGFRB	1.211933898	1.14E-07	1.13E-06
PI3K-Akt signaling pathway	JAK2	-1.391087611	7.98E-11	1.59E-09
PI3K-Akt signaling pathway	IFNAR1	-1.104670053	8.93E-07	7.44E-06
PI3K-Akt signaling pathway	ITGA2	-1.347420377	1.64E-09	2.43E-08
PI3K-Akt signaling pathway	CCNE2	-1.132772664	7.23E-11	1.45E-09
PI3K-Akt signaling pathway	GNG11	1.340623309	1.07E-20	2.01E-18
PI3K-Akt signaling pathway	CDKN1A	1.753059588	3.46E-33	5.14E-30
PI3K-Akt signaling pathway	PIK3R1	-1.238014643	2.21E-07	2.07E-06
PI3K-Akt signaling pathway	AKT3	-1.108506573	2.78E-06	2.10E-05
PI3K-Akt signaling pathway	ITGB5	1.015901552	2.13E-14	9.43E-13
PI3K-Akt signaling pathway	MYB	-1.26402982	3.25E-06	2.42E-05
PI3K-Akt signaling pathway	CREB3L3	-1.418108273	0.001086427	0.004866546
PI3K-Akt signaling pathway	SOS2	-1.531526124	6.60E-15	3.27E-13
PI3K-Akt signaling pathway	ITGA1	-1.012590945	1.42E-07	1.39E-06
PI3K-Akt signaling pathway	PRKAA1	-1.04896765	2.55E-08	2.93E-07
PI3K-Akt signaling pathway	MAP2K2	1.203248028	5.26E-16	3.50E-14
PI3K-Akt signaling pathway	GNB2	1.133595599	4.56E-09	6.11E-08
PI3K-Akt signaling pathway	CHAD	-1.372142137	0.001192116	0.00529437
PI3K-Akt signaling pathway	IL6	1.049252643	0.000439308	0.002142845
PI3K-Akt signaling pathway	PKN2	-1.803559946	2.28E-16	1.67E-14
PI3K-Akt signaling pathway	PRKAA2	-1.439063089	1.57E-13	5.67E-12
PI3K-Akt signaling pathway	PIK3CA	-1.411174231	4.71E-19	6.39E-17
PI3K-Akt signaling pathway	IL7R	2.596312835	3.95E-09	5.36E-08
PI3K-Akt signaling pathway	KITLG	-1.092598623	3.52E-08	3.92E-07
PI3K-Akt signaling pathway	COL9A2	1.902071985	2.88E-08	3.28E-07
PI3K-Akt signaling pathway	LAMB3	1.441665325	1.22E-24	4.53E-22
PI3K-Akt signaling pathway	IKBKG	1.346915819	5.05E-16	3.38E-14
PI3K-Akt signaling pathway	PDGFD	-1.054986596	0.000104396	0.000580124
PI3K-Akt signaling pathway	CREB3	1.095427685	2.77E-14	1.19E-12
PI3K-Akt signaling pathway	ITGB8	-1.430336372	7.00E-13	2.20E-11
PI3K-Akt signaling pathway	ATF2	-1.680716062	2.47E-13	8.56E-12
PI3K-Akt signaling pathway	PIK3R2	1.154260485	8.09E-08	8.35E-07
PI3K-Akt signaling pathway	LAMB2	1.123746763	8.30E-18	8.34E-16
PI3K-Akt signaling pathway	LAMC2	1.044533806	0.025579304	0.080116311
PI3K-Akt signaling pathway	SGK3	-1.153003142	2.26E-07	2.11E-06
PI3K-Akt signaling pathway	PPP2R3A	-1.194972349	3.39E-06	2.52E-05
PI3K-Akt signaling pathway	SOS1	-1.694634987	2.87E-14	1.22E-12
PI3K-Akt signaling pathway	FGF11	1.609562681	0.006751532	0.025047766
PI3K-Akt signaling pathway	THBS3	1.027788321	3.38E-13	1.14E-11

PI3K-Akt signaling pathway	EFNA4	1.238354112	1.44E-07	1.40E-06
PI3K-Akt signaling pathway	KRAS	-1.217668059	3.46E-11	7.44E-10
PI3K-Akt signaling pathway	CDK6	-1.419380183	3.12E-10	5.39E-09
PI3K-Akt signaling pathway	ITGAV	-1.029953061	2.90E-06	2.18E-05
PI3K-Akt signaling pathway	VWF	2.153719897	0.017222843	0.056809377
PI3K-Akt signaling pathway	BAD	1.195966943	1.29E-13	4.76E-12
PI3K-Akt signaling pathway	VTN	1.822527087	3.27E-26	1.55E-23
PI3K-Akt signaling pathway	RPS6KB2	1.01941029	4.91E-09	6.53E-08
PI3K-Akt signaling pathway	CCND3	1.440939072	1.63E-21	3.44E-19
PI3K-Akt signaling pathway	CREB5	-1.23068154	8.37E-05	0.000474812
PI3K-Akt signaling pathway	HSP90AA1	-1.010403399	7.74E-12	1.92E-10
PI3K-Akt signaling pathway	PPP2R1A	1.297958903	1.77E-20	3.10E-18
PI3K-Akt signaling pathway	COL2A1	1.981298349	0.037089754	0.110342903
PI3K-Akt signaling pathway	G6PC3	1.204272146	1.38E-11	3.25E-10
PI3K-Akt signaling pathway	PDGFC	-1.037497929	2.26E-10	4.02E-09
mTOR signaling pathway	RICTOR	-1.667914376	0.000220766	0.001147832
mTOR signaling pathway	WNT6	1.017974486	3.80E-06	2.80E-05
mTOR signaling pathway	LAMTOR4	1.108271819	1.54E-10	2.85E-09
mTOR signaling pathway	PIK3R1	-1.238014643	2.21E-07	2.07E-06
mTOR signaling pathway	FZD3	-1.105870527	4.28E-05	0.000257426
mTOR signaling pathway	BRAF	-1.077460489	7.49E-10	1.19E-08
mTOR signaling pathway	AKT3	-1.108506573	2.78E-06	2.10E-05
mTOR signaling pathway	SOS2	-1.531526124	6.60E-15	3.27E-13
mTOR signaling pathway	PRKAA1	-1.04896765	2.55E-08	2.93E-07
mTOR signaling pathway	MAP2K2	1.203248028	5.26E-16	3.50E-14
mTOR signaling pathway	PRKAA2	-1.439063089	1.57E-13	5.67E-12
mTOR signaling pathway	PIK3CA	-1.411174231	4.71E-19	6.39E-17
mTOR signaling pathway	AKT1S1	1.349401111	2.34E-18	2.70E-16
mTOR signaling pathway	FZD6	-1.182102521	8.40E-08	8.63E-07
mTOR signaling pathway	WNT5B	1.663297734	0.001425716	0.006216816
mTOR signaling pathway	FZD9	1.214904193	2.86E-07	2.62E-06
mTOR signaling pathway	FNIP2	-1.048499459	4.30E-05	0.000258365
mTOR signaling pathway	PIK3R2	1.154260485	8.09E-08	8.35E-07
mTOR signaling pathway	SOS1	-1.694634987	2.87E-14	1.22E-12
mTOR signaling pathway	FNIP1	-1.727095471	7.99E-15	3.90E-13
mTOR signaling pathway	KRAS	-1.217668059	3.46E-11	7.44E-10
mTOR signaling pathway	RHOA	1.074363739	4.03E-34	6.63E-31
mTOR signaling pathway	RPS6KB2	1.01941029	4.91E-09	6.53E-08
mTOR signaling pathway	LRP6	-1.459148199	7.55E-13	2.35E-11
mTOR signaling pathway	LAMTOR1	1.06353723	2.96E-11	6.47E-10
mTOR signaling pathway	CAB39L	-1.012738127	0.001962789	0.008315872
mTOR signaling pathway	CLIP1	-1.312784467	4.55E-15	2.35E-13
mTOR signaling pathway	RPS6KA3	-1.329726317	9.34E-11	1.81E-09

FDR represented for false discovery rate. Log2FoldChange represented for the fold changes of the log2 ratio of gene expression in si-THRB-DMSO to that in si-THRB-NOMAC treatment RL95-2 cells. *P* value was used to indicate the significance level of the differential gene between si-THRB-DMSO *vs* si-THRB- NOMAC treatment RL95-2 cells.
